# Spasticity as the First Manifestation of Ischaemic Lesions Involving the Cingulum

**DOI:** 10.1155/2013/534243

**Published:** 2013-11-04

**Authors:** Ivânia Alves, Vítor Tedim Cruz, Hans Peter Grebe

**Affiliations:** ^1^Departamento de Neurologia, Hospital São Sebastião, Centro Hospitalar de Entre Douro e Vouga, Rua Dr. Cândido de Pinho, 4520-211 Santa Maria da Feira, Portugal; ^2^Secção Autónoma de Ciências da Saúde, Universidade de Aveiro, Edifício 30 Campus Universitário de Santiago, 3810-193 Aveiro, Portugal

## Abstract

*Background and Purpose*. Spasticity is a positive sign of upper motor neuron syndrome that usually develops weeks after a stroke. The mechanisms that lead to its appearance are not completely understood, namely, the cortical regions whose lesion may induce spasticity. *Summary of Cases*. We report two patients with an ischaemic stroke entailing the anterior cingulate gyrus (pericallosal artery territory), who presented with acute hemiplegia and spasticity since symptom onset. Spasticity resolved within days after onset. *Conclusions*. The acute destruction of the anterior cingulate region, interrupting inhibitory projections towards lower motor centres, probably explains the acute onset of spasticity that occurred in these two patients. Further studies addressing the role of this region in acute and chronic disturbances of muscular tone are necessary.

## 1. Background and Purpose

Ischaemic damage to the corticospinal tract may induce both negative (muscle weakness, impaired coordination, motor planning, and fatigue) and positive signs (rigidity, spasticity, exaggerated deep tendon reflexes, Babinski signs, and clonus) [1]. Spasticity is only one of those positive features and it was defined by Lance as a motor disorder characterized by a velocity-dependent increase in tonic stretch reflexes [2].

Usually, spasticity is expected to develop only weeks after an acute ischaemic lesion. The mechanisms that mediate its appearance are not completely understood. It is known that some descending motor pathways (from higher centres and brainstem) indirectly influence the excitability of anterior horn cells. In particular, the dorsal reticulospinal tract, arising in ventromedial reticular formation, is the main inhibitory tract for spinal reflex activity [1, 3]. Cortical motor regions facilitate this area, augmenting the inhibitory drive down to the spinal cord. However, the cortical areas involved in this feature are mostly undiscovered [3]. 

Some patients were described, in whom spasticity appeared as an acute sign of stroke, namely after subcortical basal ganglia injury [4], but not in cortical lesions. Moreover, some case series whose aim was to describe the clinical and radiological patterns of the anterior cerebral artery infarction [5–7] point several hypertonia phenomena. In Lausanne stroke registry [6], the appearance of spasticity within less than 10 days in those with motor deficit is referred and Alonso et al. [5] and Kumral et al. [7] articles indicate that a spastic muscle tone emerged rapidly in most patients, but not as early as being one of the first manifestation of stroke.

 We describe the first two patients with acute onset of spasticity and hemiplegia due to ischaemic strokes involving the anterior cingulate gyrus. 

## 2. Summary of Cases

### 2.1. Patient 1

Female, 86 years old, previously healthy, presented after sudden onset of left motor deficit and sphincter incontinence. Previous medical history was irrelevant with a modified Rankin score of zero. On neurological examination, she had intact higher functions and a painful combined retro- and laterocollis on her left side, together with severe left hemiparesis and marked spasticity of both limbs and left Babinski sign. The left arm assumed a flexed position of the antigravitational muscles, with recruitment of forearm, wrist, metacarpophalangeal, and interphalangeal joints.

**Figure 1:**
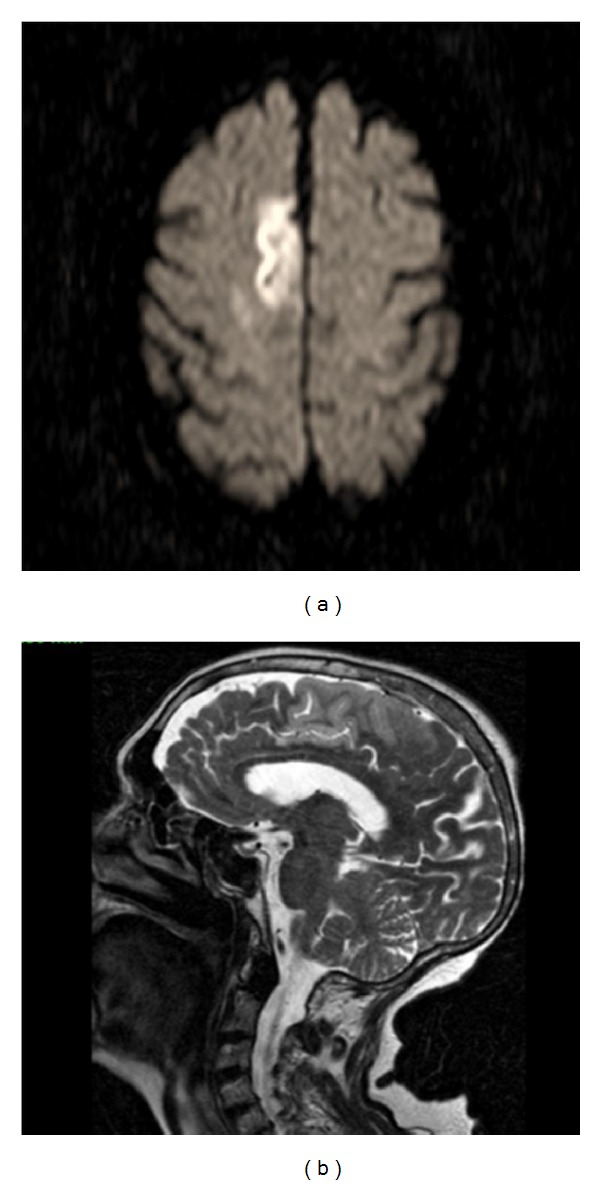
(a) Diffusion-weighted image and (b) T2-weighted image: right cortical and subcortical frontal parasagittal acute ischaemic lesion, affecting the superior frontal gyrus and the cingulate gyrus.

Brain MRI revealed an acute ischaemic lesion located on the right parasagittal frontal lobe, cortical and subcortical, but limited to the superior frontal and cingulate gyri (Figures 1(a) and 1(b)), corresponding to pericallosal artery territory. The electrocardiogram depicted atrial fibrillation. The remaining exams were normal (analysis, transcranial, carotid, vertebral, and cardiac ultrasound). Cervical MRI was normal. 

Spasticity remitted gradually over the next 48 hours and she was discharged with left flaccid hemiparesis and a modified Rankin score of four. 

### 2.2. Patient 2

Male, 55 years old, previous smoker, was admitted to the emergency department after sudden onset of left motor deficit. On neurological examination he had intact higher functions, left hemiparesis with lower limb predominance, and left Babinski sign. His left arm was very spastic, adopting a posture similar to that described in case one. As there were no contraindications, he was submitted to thrombolysis with alteplase, and an almost complete recovery ensued over the next days. 

**Figure 2:**
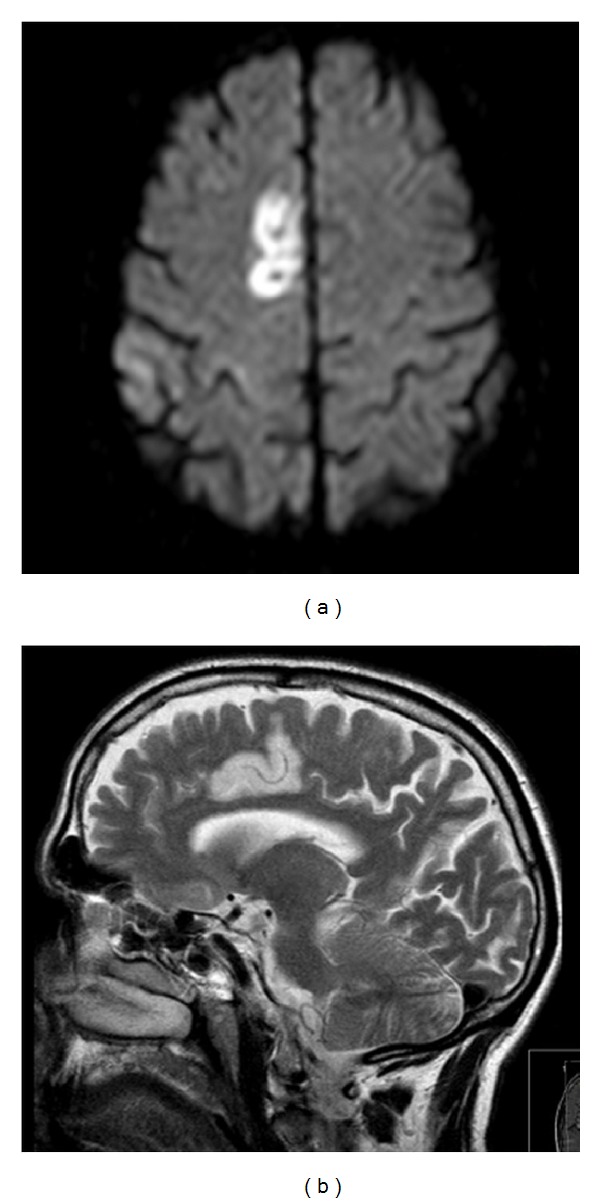
(a) Diffusion-weighted image and (b) T2-weighted image: acute ischaemic lesion on the inner surface of the frontal lobe, extending to the right cingulate gyrus.

The brain MRI showed an ischaemic lesion over the inner surface of the right frontal lobe, extending to the right cingulate gyrus (Figures 2(a) and 2(b)), corresponding also to pericallosal artery territory. There were concomitant acute ipsilateral lesions on the corona radiata and frontoopercular region. Other exams (analysis, transcranial, carotid, vertebral and cardiac ultrasound) depicted only mild hypercholesterolemia. 

The spasticity remitted in hours and the patient was discharged home, a few days later, with a modified Rankin score of one. 

## 3. Conclusion 

Although spasticity is generally expected to be a chronic manifestation of stroke, these two patients presented spasticity as a first manifestation of an ischaemic stroke. Furthermore, brain MRI revealed acute ischemic lesions located over similar areas. In patient 2 the lesion was more restricted and grossly corresponding to Brodmann area 24 [8]. 

The cingulate cortex was reported as one of the cortical suppressant regions, and several connexions with motor areas have been mentioned [9, 10]. The anterior cingulate cortex is known to be involved in error monitoring and detection, response selection, and attention control [11]. Furthermore, the anterior cingulate cortex probably contributes to perception of pain, autonomic system functioning, memory, and social and emotional interaction [10]. The dorsal cingulate sulcus has several motor regions that are active during movement, with clusters situated in dorsal midcingulate sulcus connecting to dorsal striatum, premotor cortex, and precentral gyrus. In a study that used functional brain MRI to study the cingulate region, activity in the midcingulate areas negatively predicted levels of activity in ventromedial prefrontal cortex, rostral anterior cingulate cortex, posterior cingulate cortex, and cerebellar, extrastriatal, and superior parietal regions. Specially one of the superior seeds, which was related to motor cortices (and implicated in complex motor tasks), negatively predicted activity in basal ganglia, thalamus, and superior cerebellar regions [9]. 

The acute destruction of the anterior cingulate region probably explains the acute onset of spasticity that occurred in these two patients. We can only speculate that this resulted from acute interruption of cortical inhibitory projections towards lower centres of motor control. Further studies addressing the role of this region in acute and chronic disturbances of muscular tone are necessary.
